# The odds of having obesity in Egyptian children with autism spectrum disorders is higher than stunting compared to healthy developing peers: a national survey

**DOI:** 10.1186/s12887-024-04934-5

**Published:** 2024-07-20

**Authors:** Ammal M. Metwally, Mona A. Helmy, Ahmed Aboulghate, Naglaa Abu-Mandil Hassan, Walaa S. Mahmoud, Ahmed S. Ismail, Salwa M. El Shebini, Nihad H. Ahmed, Hoda B. Mabrok, Maha H. Mahmoud, Ghada A. Elshaarawy, Amal Elsaied, Engy A. Ashaat, Amira S. ElRifay, Safaa Abdelhady, Sherif E. Eldeeb, Mostafa M. El-Saied, Sahar A. El-Masry, Nayera E Hassan, Hala Y. Badawy, Nahed A. Elghareeb, Mohamed Abdelrahman, Khadija M. Alian

**Affiliations:** 1 Community Medicine Research Department/Medical Research and Clinical Studies Institute, National Research Centre, P.O. 12622, Dokki, Cairo, Egypt; 2 Environmental and Occupational Medicine Department, Environmental and climate change Research Institute, National Research Centre, Dokki, Cairo Egypt; 3 Biological Anthropology Department/Medical Research and Clinical Studies Institute/National Research Centre, Dokki, Cairo, Egypt; 4 Nutrition and Food Science Department/Food Technology and Nutrition Division, National Research Centre, P.O: 12622, Dokki, Cairo Egypt; 5 Child with Special Needs Dept./ Medical Research and Clinical Studies Institute, National Research Centre, Dokki, Cairo Egypt; 6 Clinical Genetics Department, Human Genetics and Genome Research Institute, National Research Centre, Dokki, Cairo Egypt; 7 Child Health Department, Medical Research and Clinical Studies Institute, National Research Centre, Dokki, Cairo Egypt; 8 Department of Psychiatry, Mansoura General Hospital, Ministry of Health and Population, El Dakahlyia, Egypt; 9 Prevention of disability general directorate, Ministry of Health and Population, Cairo, Egypt; 10 Public Health and community medicine, Medical Research and Clinical Studies Institute, National Research Centre, Dokki, Giza, Egypt

**Keywords:** Autism, ASD, Nutrition, Eating, Growth status, GIT disorders

## Abstract

**Background:**

The nutritional status and growth of children with Autism spectrum disorders (ASD) is influenced significantly by two factors; food selectivity behaviors due to their consumption of a limited variety of food and the high incidence of gastrointestinal (GIT) disorders.

**Aim:**

This study aimed to assess the nutritional adequacy and growth pattern of ASD children aged three to twelve years compared to their healthy developing peers.

**Methods:**

A national comparative, facility-based cross-sectional study was conducted in eight Egyptian governorates on 285 Egyptian children diagnosed with ASD and 224 children who are their relatives as healthy developing peers. Anthropometric measurements were obtained, including weight, height, head circumference, and mid-upper arm circumference. Body Mass Index (BMI) was calculated and all numbers were plotted on WHO growth charts. Assessment of food preferences, and nutrient intake adequacy of children was done using the Food preference questionnaire, and the Dietary Reference Intakes (DRIs) of Egyptian children.

**Results:**

Calorie-dense food and sugar intake were higher among ASD children than their healthy developing peers. ASD children omit some important protein sources such as dairy (COR = 5.2, 95% CI:2.7–9.9), meat, and poultry (COR = 2.7, 95% CI: 1.6–4.7), and a lower intake of fruits and vegetables than their healthy developing peers. For children with ASD in all age groups, a deficiency in the range of 50–60% was detected for vitamins (C, D, B6, thiamine, riboflavin, niacin) and minerals (iron). A deficiency in the range of 60–70% was detected for folate and calcium. A deficiency of vitamin C calcium and iron was also detected for both children with ASD and their healthy developing relatives aged 6 to 12 years. GIT disorders were common among ASD children compared to healthy developing peers (COR = 2.8 to 10.3). Children with ASD had four-fold higher odds of stunting (COR = 4.1, CI: 1.7–10.1), threefold higher odds of being overweight (COR = 3.3, CI: 1.48–7.32), and nearly eleven-fold higher odds of obesity (COR = 11.4, CI: 4.05–32.17) compared to their healthy developing peers.

**Conclusion:**

ASD children are prone to overweight and protein malnutrition. Their intake of fruits and vegetables is inadequate and hence their intake of vitamins and minerals is insufficient, contributing to stunting.

**Supplementary Information:**

The online version contains supplementary material available at 10.1186/s12887-024-04934-5.

## Introduction

Autism spectrum disorder (ASD) is a neuro-developmental disorder characterized by three main symptoms: social interaction skills impairment, communication impairment, and repetitive as well as restrictive behaviors and interests [1]. The prevalence of ASD is estimated to be 0.06–1.4% worldwide [2].

Meanwhile, the national estimate of the prevalence of the at high risk of ASD among Egyptian children aged 1 to 12 years was 3.3% (95% CI:3.1–3.5%), with the highest prevalence for the age group 5-< 6 years (6.4%, 95% CI: 5.7–7.2%) [3]. Although autism can be diagnosed at any age, symptoms usually appear in the first two years of life [4]. Up till now the etiology of autism is Vague and not conclusive. The success of different nutritional approaches in alleviating ASD symptoms provided signals that nutrition may play an active role in the etiology of ASD [5]. At the same time, the growth and nutritional status of children with ASD are significantly influenced by two factors: food selectivity behaviors and the increased incidence of gastrointestinal tract (GIT) disorders [6–8]. Food selectivity is very frequent in ASD children and is defined simply as the consumption of an abnormally small variety of food with special refusal of intake of one or more food groups [9]. This behavior limits food choices, which, in turn, can impact nutritional status and consequently growth [10]. This behavior can be also considered a manifestation of the rigid behavior and restricted interests that characterize autism spectrum disorders [11]. Evidence also shows that GIT problems are common among children with ASD and this was linked to the existence of the gut–immune–brain pathways [12]. Common symptoms include constipation, diarrhea, nausea, and abdominal pain [13].

Having little or no communication skills and being mostly unable to tell their caregivers that they are suffering [14] makes it even more challenging to detect the distress and pain in such individuals concerning food and eating. Added to this, children with ASD might not have adequate motor skills for handling food, giving the response of fear, aggression, or escape. Also, in children with food selectivity accompanied by nausea, vomiting, or even choking, the problems could be secondary to sensory disorders. In such a case, refusal of food can be perceived as an adaptive behavioral response [14].

A few studies in Egypt and the Middle East have shed some light on the growth and nutritional status of ASD children of which some studies were done in Egypt [15, 16], Kuwait [17], and Turkey [18] .

These studies however were done on small groups, and they recommended screening of nutritional adequacy of such children. In our study, a larger sample from eight governorates with representative social and demographic aspects, comprising 285 ASD children versus 224 who are their healthy close relatives was selected to compare their eating patterns, food intake adequacy, growth, and GIT problems. The study assessed also the daily and weekly dietary intake of nutrient-rich food versus energy-dense food in ASD children compared to their peers. The study also identified the impact of the nutrient intake of ASD children on their daily recommended intake (DRI) compared to their peers.

This exploration study about dietary inadequacy intake among children with ASD was proposed to provide suggestions for future medical nutrition therapies helping in managing symptoms of the ASD population.

## Methodology

### Study design and setting

The study was a national facility-based comparative study conducted over a two years duration starting in January 2019 and ending in January 2021.

### Sampling frame and cluster preparation

A multistage cluster random sampling technique was used with three sampling frames in three stages; the first sampling frame used was the comprehensive list of the 27 governorates of Egypt, according to the enumeration census from the Central Agency for Public Mobilization and Statistics (CAPMAS) [19] within each of the four main geographic administrative regions of Egypt.

A representative sample of eight governorates was selected in the first stage to represent the main geographic areas in Egypt including one urban (Cairo), three Upper Egypt (Fayoum, Assuit, and Aswan), three Lower Egypt (Damietta, Dakahlia, and Gharbia) and one Border/Frontier (Marsa Matrouh).

In the second stage, a comprehensive list of the cities and local village units of each of the randomly chosen eight governorates of Egypt was used. Using the human development index which is based on the socio-economic status as a wealth index which was produced by the by the Economic Research Forum and CAPMS in Egypt [20], three social categories; namely low, medium, and high social class were identified. Cities (Kism) of the urban areas and local village units (Markaz) of the rural areas were listed in each social category. Three cities from urban areas and three local village units from rural areas were randomly selected from each of the chosen categories [21–23]. Phase one of this study for detected the at high risk of Autism was conducted at a community level and its results were published [3]. The list of the targeted Maternal and Child Health (MCH) and Primary Health Care (PHC) facilities according to the governorates, locality, and sociodemographic status for referring children aged three to twelve years with at high risk of ASD can be found in an additional supplementary file (S-Table 1). Referred children to MCH and PHC facilities of the Ministry of Health and Population (MOHP) were included in the current study (phase two).

### Selection of ASD children

Any referred child to the randomly selected health facilities of the MOHP in the age range of three to twelve years whether previously identified as having ASD or at high risk of Autism or with typical or deviated development according to Brown et al., 2020 [24] and WHO, 2001 [25], was screened again for ASD. Specialists from the Ministry of Health and Population screened the referred ASD children using Gilliam Autism Rating scale (GARS-2) [26]. Denver II Developmental screening test was also used for detecting developmental regression of children up to 6 years [27].

The clinical diagnosis of children suspected for autism according to GARS-2 findings, was based on the criteria of the autistic disorder as defined in the Diagnostic and Statistical Manual of Mental Disorders, Fifth Edition (DSM-5), [1] and the Childhood Autism Rating Scale (CARS) [28]. The recruited children’s health status was confirmed by specialists at the primary healthcare facilities within the randomly selected 8 governorates. All subjects diagnosed with ASD exhibited symptoms within the typical triad of autistic traits: communication impairment, social deficits, and ritualistic interests. The affirmative ASD was proved in 85% of the referred children by specialized consultants of the National Research Centre.

Exclusion criteria: Children with known or previously diagnosed genetic disorders (e.g., Down syndrome, Turner syndrome, or fragile-X syndrome) as assessed by a clinical genetics team from the National Research Centre of Egypt. The validated WHO ten-question screening tool with its probes was explained to the household for detecting any disability that is affecting hearing, vision, movement, learning, thinking, or social relationships whether for children or their parents [29, 30].

### Selection of ASD children’s healthy developing relatives

Both male and female children aged between three and twelve years, with no signs of ASD, developmental delay, or chronic diseases (celiac disease, epilepsy, diabetes, cerebral palsy, etc.) and who are close healthy developing relatives to children with ASD, were selected for the study. The recruited healthy developing peers were ASD children’s healthy relatives matched on age and socioeconomic status to the children with ASD who were enrolled in the study.

Before recruiting children (those with ASD and their healthy developing relatives), they had completed comprehensive medical and behavioral evaluations. The Arabic version of Vineland Adaptive Behavior Scales, (VABSA) was used in this study by the specialists for detecting children with deviated development or aberrant behavior who potentially had one of the neurodevelopmental disorders among which ASD was suspected. The Arabic version was validated with good reliability and validity and used in several studies in Arabic Countries Vineland Adaptive Behavior Scales, (VABSA) [31]. Denver II Developmental screening test (DDST) was used also for developmental assessment and developmental regression of children up to 6 years [27] According to the behavioral evaluation cases were assigned to one of the two groups.

Exclusion criteria for healthy developing children included any chronic medical condition influencing food intake or the use of any medications that could alter feeding.

### Sample size calculation

Group sample sizes of 254 in the autism group and 204 in their healthy developing peers group to achieve 90% power to detect a difference between the group proportions of 0.15. The proportion in group one (ASD group) is assumed to be 0.5 under the null hypothesis and 0.65 under the alternative hypothesis. The proportion in group two (their healthy developing peers) is 0.5. The proportion which is in the range of 0.48 to 0.5 for the habit Nutrient-rich foods among normal healthy children in the national survey study was the base of calculation [32, 33]. The statistics test used was the two-sided Z test with pooled variance [30, 34].

The significance level of the test was targeted at 0.05. Adding 10% expected losses due to measurement error, ensured an adequate sample size of 280 for the ASD group and 224 for their healthy developing peers’ group.

To have a representative sample covering each governorate, 35 eligible children were randomly selected by systematic random sample out of those detected and confirmed to have ASD. Cases were enrolled from eight governorates to include different patterns of food selectivity. To achieve 224 healthy developing relatives for ASD children, 285 children with ASD were enrolled.

### Nutritional and growth status assessment

Growth assessment best defines the health and nutritional status of a child because disturbances in health and nutrition, regardless of their etiology, invariably affect child growth. Anthropometric indices provide measures of nutritional status based on measures of body size relative to their distribution in a reference population. Anthropometric indicators measure achieved nutritional status rather than nutrition inputs, are less subject to measurement error than dietary intake data, and also are less expensive. Nutritional status assessment was carried out through Anthropometric measurements of (weight and height) and mid-arm circumferences which are effective methods for evaluating dietary intake, growth status, and nutritional status in children with ASD.

All measurements were made according to techniques described in the anthropometric standardization reference manual [35]. Height was measured to the nearest 0.1 cm using a Holtain portable anthropometer. Weight was determined to the nearest 0.01 kg using a “Seca Scale Balance” with the subject dressed in minimal clothes and without shoes. The BMI was calculated as weight (in kilograms) divided by height (in meters) squared. All scores were calculated based on the WHO growth standards with the help of the Anthro-Program of PC [36].

To allow a comparison of growth with the WHO and growth curves, z scores (WAZ, HAZ, and WHZ) were calculated for each child. They were measured, following the recommendations of the “International Biological Program” [36–38] Height-for-age is used to indicate past nutritional deficits as it reflects a child’s nutritional history.

### Dietary pattern assessment

Qualitative information about the different items of food and beverages consumed by children was done. The dietary pattern was used to assess preferences, availability of different food items, and dietary habits of children. The food preference questionnaire was used as it is based on foods commonly eaten in Egypt. The frequency of food eaten per week, which is called share of the stomach was determined.

Parents were asked to fill out a food inventory by reporting whether or not their children would eat each of the listed foods from twelve food groups. The food groups are vegetables, fruits, dairy products, meat and poultry, fish, eggs, grains, cereals, beans, unhealthy food (desserts, and fast food), healthy beverages, and sugar-sweetened beverages that are commonly consumed in the Egyptian diet. Information about the food frequency intake of children was also collected. The daily intake for the food groups was obtained by summing the foods accepted by children within each of the food groups. This measure was used as a measure of food selectivity. Parents were asked to report about their child’s typical dietary intake using a one-day food recall diary. The frequency intake of all food items were assessed according to Ferguson et al. 1995, including; Fruits, Vegetable, Dietary fiber, Fat, Dairy products, meat and poultry, fish, eggs, grains, cereals, beans, unhealthy food and beverage. Ferguson and his colleagues’ technique has been used for assessing the intakes of rural Malawian women with similar context to that of the Egyptian women [39].

The intake of different diversity of nutrient-rich foods (vegetables, fruits, and nutrient-rich sources of protein and dairy products) versus calorie-dense foods with low levels of nutrients (French fries, chips, sugar-sweetened beverages (SSB), biscuits, chocolate, pancakes/cakes, ice cream, and candies) was compared for weekly consumption. The diversity of nutrient-rich foods eaten per week was measured as an indication of the weekly share of the stomach [40]. An increase in the number of types of nutrient-rich foods overall and per food group that was eaten per week is considered an indication of a good share of the stomach from all the plates eaten every day. The daily share of the plate as per the served meal was measured (energy-dense food in relation to the nutrient-rich food).

Dietary recalls were entered into the Egyptian nutrient calculating software, to calculate macro- and micro-nutrient profiles. The prevalence of nutrient adequacy was assessed using the Dietary Reference Intakes (DRIs) [41, 42] to calculate percent relative to the Recommended Dietary Allowance (RDA). Nutritional inadequacy was defined as the percentage of children in each group who did not meet the DRIs for the specific age and sex. A detailed description of all food and beverages consumed was recorded. Individual food intake was calculated and analyzed using Computer Aided Nutritional Analysis Program, the food composition Table 2006, National Nutrition Institute, Cairo, Egypt [43].

The Recommended Dietary Allowance (RDA) for micronutrients and vitamins was calculated according to Food and Agriculture Organization (FAO)/WHO 2002 [44] and the RDA for energy and macronutrient according to FAO/WHO/United Nations University 2004 (FAO/WHO/UNU 2004) [44].

### Gastrointestinal (GI) disorders assessment

Assessing changes in GI disorders: The GI Severity symptoms were assessed for four symptoms; appetite disorder, constipation, diarrhea and abdominal pain. The used questionnaire was aiming to measure the severity of gastrointestinal discomfort over the previous week of each visit. Parents of the study participants were requested to document a record on their children’s diary cards for any of the mentioned GI discomforts on a daily basis over a week. Accordingly, the GI symptoms were determined from the parents’ diary records.

### Survey implementation

Before the implementation of the current facility-based phase of the study, a community house-to-house phase was done as a screening for children with developmental delay including those at high risk of autism for the 8 randomly selected governorates of Egypt (S-Table 1). VABS survey form was administered by trained social workers who were provided with appropriate training in interview techniques according to the National Research Council, 2002. A delay in a specific domain of VABS is considered if the score of that domain is 2 SD below the mean (< 70). The VABS survey form demonstrated a high-reliability coefficient ranging from 0.83 to 0.94 across all domains according to a number of old [45] and recent studies [46, 47] used for verifying autistic social dysfunction. Discovered children with low scores (< 70) on VABS was suggestive for developmental delay (DD) according to Sparrow and his colleagues [48] .These suspected children for DD were referred to maternal and child health (MCH) centers as well as the primary health care (PHC) centers of the Egyptian Ministry of Health and Population (MOHP). All referred suspected cases with delay were screened for ASD using ASD screening tools [27, 41] as well as the confirmatory tools [1, 28]. Whereas, the close relatives of the diagnosed and referred children with autism who were matched for age were invited to MCH centers to be assessed using VABS [31] and DDST [27] as described before.

This manuscript is a piece of work that is derived from a National population for assessment of the prevalence of developmental delays [49–51] disabilities [52, 53] as well as autism [3] among children aged 1 to 12 years. All children with detected disabilities or delay other than autism are referred to rehabilitation centers of the Egyptian MOHP for management. Children with ASD were referred to the clinic of the Clinical Genetics Department and outpatient clinics of Children with Special Needs of the National Research Centre of Egypt for management.

### Statistical analysis

The collected data was reviewed for its completeness and accuracy and was statistically analyzed. Data were analyzed using Statistical Package for the Social Sciences (SPSS) version 24.0 software [54]. All data were represented by mean (SD) and percentages. Comparison between groups was done by the following statistical tests: the Student’s t-test to compare socio-demographic data and the chi-squared (x2) test to assess the statistical significance of differences among categorical data. The nonparametric Fishers exact test (two-tailed) was used instead of the chi-squared test in cases of very small sample sizes. Z test was used between the two proportions. Unpaired t-tests were used to compare the differences between two means for ASD with their healthy developing peers. The crude odds ratios (COR) [55] and 95% confidence intervals (CI) were calculated in comparison between ASD and their healthy developing peers. A significant association is considered if the 95% CI does not include the value 1.0, and a cutoff p-value of less than 0.05 is used for all tests of statistical significance in this study. Probability values (*P*) of less than 0.05 were regarded as statistically significant.

## Results

Demographic data of the studied children with ASD and their healthy developing peers is illustrated in Table 1. Participants were slightly higher in urban communities than in rural ones. They were equally distributed across social classes. Both groups have comparable ages for their mothers and fathers. Regarding education levels, the majority of fathers and mothers had completed their secondary school. The only demographic variable that was different between both groups was parents’ consanguinity, being significantly higher in children with ASD.

**Table 1:** Sociodemographic characteristics of the studied children with ASD and their healthy developing peers

Sociodemographic characteristics	Children with ASD *n* = 285 *n* (%)	Healthy developing peers *n* = 224 *n* (%)	Test of Sig
**Social Class**			
Low (*n*, %)	73(25.6)	69(30.8)	χ ^2^ = 1.74, *P* = 0.42
Middle (*n*, %)	104(36.5)	78(34.8)	
High (*n*, %)	108 (37.9)	77(34.4)	
**Urban/ Rural**			χ ^2^ = 1.38, *P* = 0.24
Urban (n, %)	165(57.9)	118(52.7)	
Rural (*n*, %)	120(42.1)	106(47.3)	
**Age of children**			
Mean ± SD	6.6 ± 2.5	6.8 ± 2.5	t = 0.90, *P* = 0.37
< 6 years (*n*, %)	114(40.0)	98(43.8)	χ ^2^ = 0.72, *P* = 0.39
> 6–12 years (*n*, %)	171(60.0)	126(56.3)	
**Mother age: Mean ± SD**	26.75 ± 5.9	26.08 ± 6.05	t = 1.26, *P* = 0.21
**Father age: Mean ± SD**	33.34 ± 6.8	32.84 ± 7.76	t = 0.77, *P* = 0.44
**Presence of history of consanguinity (n**,** %)**			
1st Degree 2nd Degree No Consanguinity	89(31.2)	32(14.3)	χ ^2^ = 23.8, *P* < 0.001**
	51(17.9)	34 (15.2)	
	145(50.9)	158(70.5)	
**Maternal Education**			
Preparatory schools or less	88(30.9)	84(37.5)	χ ^2^ = 2.46, *P* = 0.12
Secondary schools or above	197(69.1)	140(62.5)	
**Paternal Education**			χ ^2^ = 0.72, *P* = 0.40
Preparatory schools or less	67(23.5)	60 (26.8)	
Secondary schools or above	218(76.5)	164(73.2)	

**=*p*-value highly sig at < 0.01

A comparison of food selectivity and preferences between children with ASD and their peers was assessed using food frequency questionnaire (FFQ) with twelve food groups: vegetables, fruits, dairy products, meat and poultry, fish, eggs, grains, cereals, beans, unhealthy food (desserts, and fast food), healthy beverage, and sugar-sweetened beverages as shown in Table 2.

Food selectivity in 285 children with ASD was compared to that of 224 healthy developing children of their relatives. Across all food groups, the percentage of children with ASD who omitted types of foods was higher than those without ASD according to their parents’ reports. Children with ASD omitted significantly the dairy group of food by nearly five-fold than their healthy developing peers (COR = 5.2, 95% CI: 2.7–9.9). ASD children omitted meat and poultry group two and a half times higher than their peers (COR = 2.7, 95% CI: 1.6–4.7). Children with ASD also tended to have a significantly very low preference for fish and eggs (COR = 1.95, 95% CI: 1.37–2.78 and COR = 1.5, 95% CI: 1.1–2.18 respectively). There was an insignificant difference regarding the intake of grains, vegetables, cereals, and fruits.

The three most preferred vegetables were potatoes, cucumber, and vegetable salads. Their intake of rice (COR = 2.28, 95% CI:1.58–3.28), chips (COR = 1.5, 95% CI:1.04–2.15) and sugar-sweetened beverages (COR = 1.8, 95% CI:1.21–2.70) was significantly higher than that of their healthy developing peers. Carbonated soft drinks and tea were the top choices for ASD children. Although cheddar and cream cheese and omelet were among ASD children’s preferred items, their intake was significantly less than that consumed by their healthy developing peers (COR = 0.38, 95% CI: 0.26–0.55 and COR = 0.35, 95% CI: 0.24–0.5 respectively).

**Table 2:** Common food groups omitted and preferred by children with ASD compared to their healthy developing peers

Food groups	Children with ASD *n* = 285 *n* (%)	Healthy developing peers *n* = 224 *n* (%)	COR (CI)
**Food group omitted**			
Vegetables	6 (2.1)	0 (0.0)	4.79 (0.57–40.13)
Fruits	22 (7.7)	8 (3.5)	2.26 (0.99–5.17)
Dairy*	65 (22.8)	12 (5.4)	5.22 (2.74–9.94)**
Meat and poultry*	63 (22.1)	21 (9.3)	2.74 (1.62–4.66)**
Eggs*	119 (41.7)	72 (32.1)	1.51(1.05–2.18)*
Grains	0 (0.0)	5 (2.2)	0.15 (0.018–1.33)
Fish*	173 (60.7)	99 (44.1)	1.95 (1.37–2.78)*
Cereals	12 (4.2)	8 (3.5)	1.19 (0.49–2.95)
Beans*	41 (14.4)	9 (4.01)	4.01 (1.91–8.45)**
Unhealthy food (fast food, candies, French fries, fried noodles, pancakes/cakes)*	28 (9.8)	19 (8.5)	1.18 (0.64–2.17)
Healthy drinks (milk, herbal beverages, fresh home made juice)	135 (47.4)	92 (41.1)	1.29 (0.91–1.84)
Sugar Sweetened beverages (canned juice, carbonated soft drinks)	141 (49.5)	120 (53.6)	0.85 (0.59–1.2)
**Most frequent preferred food items eaten > 3 times /week per food groups**			
Vegetables (salad: cucumber/lettuce/parsley/onion)	45 (15.8)	50 (22.3)	0.65 (0.42–1.02)
Fruits (Banana)	52 (18.3)	34 (15.2)	1.25 (0.78-2)
Dairy (Yoghurt)	80 (28.0)	50 (22.3)	1.36 (0.9–2.04)
Dairy (Cheese slice, cheddar, and cream)*	74 (26.0 )	108 (48.2)	0.38 (0.26–0.55)**
Eggs (omelette egg)	132 (46.3)	160 (56.1)	0.35 (0.24–0.5)**
Meat and poultry (beef and lamb)	68 (23.8)	68 (30.3)	0.72 (0.48–1.07)
Cereals (rice)	145 (50.8)	70 (31.3)	2.28(1.58–3.28)**
Cereals (pasta)	94 (33.0)	85 (37.9)	0.80(0.56–1.60)
Grains (white bread)	139 (48.7)	124 (55.4)	0.76(0.54–1.09)
Desserts (biscuits and crackers)	93 (32.6)	58 (25.8)	1.39 (0.94–2.04)
Unhealthy (chips)	131 (45.9)	81 (36.2)	1.5 (1.04–2.15)*
Sweetened (rice pudding)	43 (15.0)	25 (11.1)	1.41(0.83–2.4)
Unhealthy (French fries)	116 (40.7)	84 (37.5)	1.14 (0.79–1.63)
Beans	103 (36.1)	95 (42.4)	0.73(0.51–1.05)
Sugar sweetened beverages(Carbonated soft drink)	94 (33.0)	48 (21.4)	1.80 (1.21–2.70)*
Beverages (Tea )	48 (16.8)	50 (22.3)	0.7 (0.45–1.09)

*= *p*-value significant at < 0.05, **=*p*-value highly sig at < 0.01

Considering the weekly share of the stomach, more than half (60.3%) of the food consumed by healthy children was nutrient-rich, due to the consumption of dairy and beans/meat versus *45.3% for children with ASD as shown in* Table 3.

The percentage of energy-dense food intake per all day’s meals (Share of the plate) carried more than double the odds for children with ASD than their peers, with the highest odds for breakfast (COR = 2.9, 95% CI = 1.8–4.5) and snacks between meals (COR = 2.9, 95% CI = 1.9–4.4).

**Table 3:** Comparison between children with ASD and their healthy developing peers on their weekly and daily dietary intake of nutrients - rich food versus energy- dense food

Parameters	Children with ASD, *n* = 285 *n* (%)	Healthy developing peers, *n* = 224 *n* (%)	Crude Odds (CI)§
**Diversity of nutrient- rich foods (vegetables, fruits, nutrient rich sources of protein and dairy products) eaten per week**			
Energy-dense food Nutrient-rich foods (overall) Carbohydrates/grains	68 (23.86%) 129 (45.26%) 88 (30.88%)	33 (14.7%) 135 (60.3%) 56 (25%)	2.2 (1.3–3.5) **
**Share of the stomach (Frequency of nutrient-rich foods as per food groups eaten per week)**			**Chi square#**
Dairy Meat/beans/eggs Fruits Vegetables	13 (4.6%) 55(19.3%) 37 (13.0%) 24 (8.4%)	32 (14.3%) 52 (23.2%) 33 (14.7% 18 (8.0	0.111
**Share of the Plate as per meals (daily)**			COR (CI)
*Breakfast* Energy-dense food Nutrient-rich foods (overall) Carbohydrates/grains	106 (37.2%) 119 (41.8%) 60 (21.1%)	36 (16.1%) 116 (51.8%) 72 (32.1%)	2.9 (1.8–4.5) **
*Lunch*			2.1 (1.2–3.5) **
Energy-dense food Nutrient-rich foods (overall) Carbohydrates/grains	60 (21.1%) 140 (49.1%) 85 (29.8%)	25 (11.2%) 120 (53.6%) 79 (35.3%)	
*Dinner* Energy-dense food Nutrient-rich foods (overall) Carbohydrates/grains	89 (31.2%) 154 (54. 1%) 42 (14.7%)	43 (19.2%) 132 (58.9%) 49 (21.9%)	1.8 (1.2–2.7) *
*Snack/anything between meals*			2.9 (1.9–4.4) **
Energy-dense food Nutrient-rich foods (overall) Carbohydrates/grains	156 (54.7%) 54 (18.9%) 75 (26.4%)	92 (41.1%) 92 (41.1%) 40 (17.8%)	

§ Odds calculated for Energy dense vs. nutrient rich between children with ASD and healthy developing children, # Chi square between children with ASD and healthy developing children

*= *p*-value significant at < 0.05, **=*p*-value highly sig at < 0.01

Table 4 shows the mean daily nutrient intake and the percent relation to the RDAs/DRI of children with ASD and their healthy developing relatives. The adequacy of the daily nutrient of ASD children was compared with their healthy developing peers. The adequacy was assessed using the Dietary Reference Intakes (DRIs) for calculating percent relative to the Recommended Dietary Allowance (RDA).

All the daily vitamins and minerals intake for children with ASD showed inadequacy as they were lower than their RDAs/DRI compared to their close relatives. However, the intake of the two major macronutrients (fat and carbohydrates) was insignificantly higher than their healthy developing relatives. Children with ASD had insignificantly lower intake of protein compared to their relatives.

In this context, data shows a severe deficiency (< 50%) of some important nutrients such as vitamin C and B12 and zinc for children with ASD and their healthy developing relatives. For children with ASD in all age groups, a deficiency in the range from 50 to 60% was detected for vitamins (D, B6, thiamine, riboflavin, niacin) and minerals (iron). A deficiency in the range of 60–70% was detected for minerals (folate and calcium). A deficiency of Vitamin C was observed for both groups. A deficiency of calcium and iron was also detected for both children with ASD and their healthy developing relatives aged 6 to 12 years.

**Table 4:** Comparison between the mean daily nutrients intake and their adequacy (% of RDA) of children with ASD versus their healthy developing peers

	< 6 years			> 6–12 years		
Nutrient intake (t test)§	Children with ASD *n* = 114	Healthy developing peers *n* = 98	RDA /DRI	Children with ASD *n* = 171	Healthy developing peers *n* = 126	RDA /DRI
	**Mean ± S E (%RDAs)**		**RDA /DRI**	**Mean ± S E (%RDAs)**		RDA /DRI
Energy (kcal)	958.47 ± 12.41 53.24%	891.01 ± 42.30 59.40%	1800	1357.27 ± 21.60 67.86%	1212.14 ± 27.11 60.6%	2000
t test	< 0.001**			< 0.001**		
Protein (g)	19.26 ± 4.16 80.25%	21.07 ± 3.25 87.8%	24 g	30.18 ± 6.20 67.07%	31.85 ± 6.25 70.8%	45 g
t test	0.1			0.207		
Fats (g)	28.51 ± 7.23 73.10%	20.69 ± 4.21 53.1%	39 g	49.67 ± 9.57 80.11%	49.74 ± 5.37 80.1%	62 g
t test	< 0.001**			0.96		
Carbohydrates (g)	156.21 ± 12. 03 78.11%	155.13 ± 11.42 77.56%	200 g	197.38 ± 22.34 87.72%	159.27 ± 29.16 70.79%	225 g
t test	0.358			< 0.001**		
Vitamin A (µg RE)	261.94 ± 56.33 52.4%	275.31 ± 21.98 55.06%	500	363.41 ± 36.17 51.91%	379.34 ± 22.29 54.2%	700
t test	< 0.001**			< 0.001**		
Vitamin C (mg)	10.50 ± 1.61 23.3%	24.68 ± 0.43 54.84%	45	21.61 ± 2.11 48.0%	22.80 ± 0.35 50.67%	45
t test	< 0.001**			< 0.001**		
Vitamin D (mcg)	2.86 ± 0.65 57.20%	6.95 ± 2.49 139%	5	2.70 ± 0.23 54.00%	6.52 ± 2.07 130.4%	5
t test	0.008**			0.003**		
VitaminB6 (mg)	0.33 ± 0.03 55.00%	0.49 ± 0.22 81.7%	0.6	0.42 ± 0.30 42.00%	0.73 ± 0.22 73.0%	1
t test	0.577			0.809		
VitaminB12 (mcg)	0.40 ± 0.02 33.33	0.95 ± 0.17 79.2%	1.2	0.79 ± 0.21 43.89%	1.28 ± 0.34 71.1%	1.8
t test	< 0.001**			0.2		
Thiamine (mg)	0.31 ± 0.01 51.67%	0.56 ± 0.17 93.3%	0.6	0. 49 ± 0.14 54.44%	0.67 ± 0.30 74.4	0.9
t test	0.511			0.11		
Riboflavin (mg)	0.35 ± 0.04 58.33%	0.50 ± 0.68 83.3%	0.6	0.52 ± 0.31 57.78%	0.75 ± 0.28 83.3%	0.9
t test	0.865			0.601		
Niacin (mg)	4.32 ± 1.27 54.00%	6.77 ± 1.83 84.6%	8	6.72 ± 1.43 56.00%	9.97 ± 1.83 83.1%	12
t test	0.004**			< 0.001**		
Folate(mg)	121.15 ± 11.24 60.58%	180.25 ± 25.52 90.1%	200	159.19 ± 20.03 53.06%	255.25 ± 25.12 85.1%	300
t test	< 0.001**			< 0.001**		
Calcium (mg)	501.72 ± 28.21 *62.72%*	714.97 ± 12.01 89.37%	800	650.27 ± 22.31 54.19%	765.97 ± 22.01 63.8	1200
t test	< 0.001**			< 0.001**		
Iron (mg)	5.85 ± 1.61 58.50%	7.09 ± 1.41 70.9%	10	5.60 ± 1.30 56.00%	5.68 ± 1.29 56.8%	10
t test	0.084			0.892		
zinc (mg)	3.12 ± 1.39 31.2%	4.30 ± 2.64 43.0%	10	4.12 ± 0.31 41.20%	4.78 ± 0.09 47.8%	10
t test	0.227			< 0.001**		

§ t test between 2 means, *= *p*-value significant at < 0.05, **=*p*-value highly sig at < 0.01

Children with ASD had ten times higher odds of having constipation than their healthy developing peers (COR = 10.32, 95% *CI*: 5.06-21.079). They carried also more than four times the odds to have appetite disorders (COR = 4.02, 95% CI: 2.6–6.7) and diarrhea (COR = 4.24, 95% *CI*: 2.02–8.91). ASD children had two times higher odds for pain than their healthy developing peers (COR = 2.81, 95% *CI*: 1.3–6.04) as shown in Table 5.

**Table 5:** Percentage distribution of gastrointestinal disorders among children with ASD versus their healthy developing peers

Gastro-intestinal disorders	Children with ASD, *n* = 285 *n* (%)	Healthy developing peers, *n* = 224 *n* (%)	Crude Odds (CI)
**Appetite disorders**			
Yes	124 (43.5%)	36 (16.1%)	4.02 (2.6–6.16)**
No	161 (56.5%)	188 (83.9%)	
**Constipation**			10.32 (5.06–21.07)**
Yes	86 (30.2%)	9 (4.0%)	
No	199 (69.8%)	215 (96.0%)	
**Diarrhea**			
Yes	43 (15.1%)	9 (4.0%)	4.24 (2.02–8.91)**
No	242 (84.9%)	215 (96.0%)	
**abdominal pain (stomach problems**			
Yes	30 (10.5%)	9 (4.0%)	2.81 (1.3–6.04)*
No	255 (89.5%)	215 (96.0%)	

*= *p*-value significant at < 0.05, **=*p*-value highly sig at < 0.01

The anthropometric data of participants is presented in Table 6. The majority of children were classified as having a healthy weight and height in both groups as detected by the BMI z-scores, weight for height z- scores, and weight for age z- scores. Children with ASD had four-fold higher odds of stunting (COR = 4.1, 95% *CI*: 1.7–10.1), threefold higher odds of being overweight (COR = 3.3, 95% *CI*: 1.48–7.32), and nearly eleven-fold higher odds of obesity (COR = 11.4, 95% *CI*: 4.05–32.17) compared to their healthy developing peers. On the other hand, wasting did not show a statistically significant difference between both groups (COR = 0.88, 95% *CI*: 0.33–2.32).

The pattern of distribution of underweight, stunting, wasting, overweight, and obesity was the same between males and females of all children enrolled in the study with slightly more percentages among males than females in both groups.

Children with ASD, when compared to their healthy developing peers, showed significantly lower head circumference for all children with ASD (*p* = 0.01), while their mid-upper arm was significantly higher than that of their healthy developing peers aged less than six years.

**Table 6:** Comparison between the growth indices of the studied children with ASD versus their healthy developing peers

Variables	Children with ASD, *n* = 285, *n* (%)	Healthy developing peers *n* = 224, *n* (%)	Test of Sig. *P* value
**WAZ**,** Underweight**			
Male	4(1.4%)	1(0.5%)	z = 1.09 *P* = 0.28
Female	1(0.4%)	0	
Total**+**	5(1.8%)	1(0.5%)	
COR (CI) **§**	0.03 (0.01–0.8), Insig.		
**HAZ**,** Stunting**			
Male	26(9.1%)	4(1.8%)	χ ^2^ = 2.14 *P* = 0.14
Female	3(1.1%)	2(0.9%)	
Total**+**	29(10.2%)	6(2.7%)	
COR (CI) **§**	4.1 (1.7–10.1)*, Sig.		
**WHZ**			
**Wasting**			
Male	9(3.2%)	7(3.1%)	z = 0.02 *P* = 0.98
Female	0	1(0.5%)	
Total**+**	9(3.2%)	8(3.8%)	
COR (CI) **§**	0.88 (0.33–2.32), Insig.		
**Overweight**			
Male	26(9.1%)	5(2.2%)	χ ^2^ = 1.78 *P* = 0.18
Female	5(1.8%)	3(1.3%)	
Total**+**	31(10.9%)	8(3.6%)	
COR (CI) **§**	3.30 (1.48–7.32)*, Sig.		
**Obese**			
Male	41(14.4%)	2(0.9%)	χ ^2^ = 2.74 *P* = 0.10
Female	8(2.8%)	2(0.9%)	
Total**+**	49(17.2%)	4(1.8%)	
COR (CI) **§**	11.4 (4.05–32.17)*, Sig		
	**Mean ± SD**	**Mean + SD**	
Weight/height z-score	1.11 ± 1.89	0.28 ± 0.83	Z = 5.47 *P* = 0.00**
Height/age z-score	0.59 ± 1.19	0.94 ± 1.04	Z = 4.9 *P* = 0.00**
Weight/age z-score	0.72 ± 1.32	0.29 ± 0.61	Z = 1.97 *P* = 0.049*
BMI/age z-score	0.78 ± 1.38	0.27 ± 0.93	Z = 7.31 *P* = 0.05*
Head circumference (cm) ≤ 6 years > 6- years	50.74 ± 2.43 52.61 ± 2.55	57.41 ± 3.8 63.77 ± 7.95	t = 13.39 *P* = 0.00** t = 9.39 *P* = 0.00**
MUC circumference (cm) ≤ 6 years > 6- years	17.53 ± 3.22 20.63 ± 4.78	16.29 ± 2.48 19.14 ± 3.78	t = 3.003 *P* = 0.003** t = 2.21 *P* = 0.03*

WAZ = Weight-for-age Z score, HAZ = Height-for-age Z score, WHZ = Weight-for- Height Z score, § odds for children with ASD with the condition in relation to their healthy developing peers, *= *p*-value significant at < 0.05, **=*p*-value highly sig at < 0.01

## Discussion

Our study identified and compared the food preferences, growth indices, and gastrointestinal conditions of 285 Egyptian children confirmed with ASD versus 224 who are their healthy close relatives. The enrolled children aged 3–12 years old and were selected from eight governorates of Egypt.

For a variety of reasons, children with ASD may not get the adequate nutrition they need for their healthy growth and development. Some children with ASD eat certain foods because of how the foods feel in their mouths, others might avoid eating certain foods because they associate them with discomfort or abdominal pain. Other children with ASD may be restricted to limited diets for reducing ASD symptoms [56].

Across all food groups, the percentage of children with ASD who omitted types of foods was higher than their healthy developing peers. We can see that children with ASD did not reach the RDA due to their refusal and limited food choices from all types of food groups “their food selectivity”. This selectivity could result from the GIT symptoms, the abnormal appetite, and limiting the daily and weekly intake of nutrient-rich food that was observed in the current study. Moreover, although a few children with ASD omitted vegetables and fruits, they still ate fewer fruits and vegetables than their healthy developing peers, and their intake did not exceed 50%. This resulted in both micronutrient and macronutrient (protein and healthy fats) deficiencies with their harmful sequelae. Patricia et al., (2019) [57] reported a limited intake of dairy proteins together with calcium and vitamin D inadequacy, with increased intake of other protein types in ASD children. They reported as well, higher intakes of fruits and vegetables which are similar to our results. We found in the current study that all the daily vitamins and minerals intake for children with ASD were lower than their close healthy developing relatives. Meanwhile, a higher intake of carbohydrates and a lower intake of protein were insignificantly detected among children with ASD compared to their peers. Protein, calcium, and vitamin D are major contributors to bone growth and health, thus the suboptimal skeletal growth in the current study could be explained by the insufficient intake of these elements. In support of our study, Arija et al., 2023 [58] found that all ASD children had a high intake of energy-dense foods (sugars and fats). In the current study, a very highly inadequate intake of vitamins (C, B12) and iron (< 50) and a moderately inadequate intake (50–60%) were detected for vitamins (D, B6, thiamine, riboflavin, niacin) and iron. Again, another recent research [59] supporting our results stated there is a consensus that children with ASD have selective eating patterns, food neophobia (fear of trying new foods), limited food repertoire, and sensory issues, with inadequate micronutrient intake.

GIT manifestations are frequently reported in the literature as an association with ASDs [11, 60].

Appetite abnormality is well known among children with ASD and may be attributed to micronutrient deficiencies (iron and zinc), or some medications used with autism [11, 60]. Some of them can reduce the amount of food eaten; others may increase appetite or affect the absorption of certain vitamins and minerals which in turn affect the growth of the child and again contribute to stunting or obesity. The first explanation works well with the funding of the current study in which severe deficiency with the intake of less than 50% of RDI was detected for VIT C, VIT B12, and zinc for all children with ASD and for VIT B6 for children with ASD aged 6–12 years. The observed frequent gastrointestinal complaints that carried significantly higher odds among children with ASD than their healthy developing peers in the current study especially constipation followed by appetite disorders, diarrhea then abdominal pain in the current study could be considered as an important factor that affects the eating behavior of these children. These symptoms could be also the cause or the result of food selectivity: either by being genuinely related to the autistic manifestations, preventing the child from certain food intake, like dairy products, or they could be the result of excess specific foods like sugars and starches consumed because of the abnormal (selective) autistic behavior.

Comparison of anthropometric data between the two groups as an outcome of their dietary intake and gastrointestinal disorders showed significantly higher BMI z-scores, weight for height z-scores, and weight for age z-scores for children with ASD: meaning a higher prevalence of overweight and obesity in ASD subjects than their healthy developing peers. This could be explained by the high intake of grains, potatoes, cereals and fruits of children with ASD detected in the current study. It was found that the most frequently preferred and eaten food items more than 3 times per week and more than that of their peers were in order: rice (50.8%), chips (45.9%), French fries (40.7%) then biscuits and crackers (32.6%). So, the food items preferred by these children were foods that are high in sugar content, as well as energy-dense food that was twice more than their healthy developing peers, with a less weekly share of the stomach to nutrient-rich foods compared to their healthy developing peers. Comparing our results to other research studies in different countries, they were consistent revealing a high prevalence of overweight children with ASD [15, 57, 61]. Also, a meta-analysis systematic review by Zhen et al., 2017 [62] showed a significant association between obesity and ASD and stated that children with ASD may be more prone to overweight and obesity. Thus, research in this field supports our findings and sheds light on this prevalent problem in ASD patients. Generally speaking, overweight Egyptian children are more at school age as they eat a narrow diversity of nutrient-rich food and more calorie-dense foods, every day [32], a condition that improved too much with the implementation of the national school feeding programs in Egypt [63].

In fact, the intake of healthy food is a challenging problem that starts with faulty weaning practices with a tendency to have nearly 50% overweight and obese children, especially in resource-poor settings of Egypt [64].

On the other hand, height z-scores were significantly lower in the ASD group, with no absolute stunting. This could be attributed to the detected micronutrient deficiencies because of the less-varied diet that was noticed among the ASD group compared to their healthy developing peers, presenting a food-selective behavior. Following our results, many researchers suggested food selectivity to be more common in children with ASDs than in typically developing children, and that a limited food repertoire may be accompanied by nutrient inadequacies [6, 65] as well as micronutrient deficiencies [66, 67]. Meanwhile, not all studies revealed this lower height Z-scores, one study showed insignificantly taller ADS children in younger age groups [68]. These inconsistencies might have resulted from the differences in study design.

Concerning mid-upper arm circumference (MUAC), an important tool for assessing nutritional status, it tended to be higher in ASD subjects than their healthy developing peers, with significant values in older groups. MUAC is a measure of the sum of the muscle and subcutaneous fat in the upper arm. In severe malnutrition, both fat and muscle are reduced in the upper arm [69]. Also, it reflects body fat storage, meaning possibly a larger body fat percentage in ASD subjects, harmonizing with other findings of overweight prevalence among ASD children, both resulting from excess carbohydrate intake. Our results came following a study done by Samir and Patil, 2018 [70] that compared the nutritional status of children with ASD and normal children and found a higher mid-arm circumference in the ASD group, together with a tendency to be overweight and obese.

The head circumference (HC) z scores were found to be significantly lower in children with ASD, possibly due to insufficient protein intake and micronutrient deficiencies detected among ASD children as reported in our results. We assume the same cause for both decreased height and head circumference, as they both reflect skeletal growth and could be affected by micronutrient deficiencies and protein inadequacy. Variable results were detected by other studies [71, 72].

These differences may be because of the other multiple factors contributing to head size like maternal nutrient deficiencies during pregnancy, breastfeeding [73], genetic factors, and pathological factors related to the underlying condition causing autism. Nicolaou and his colleagues, 2020 [74] stated that the main risk factors associated with HC are similar to those associated with body length. This finding is consistent with the current study and with our assumption as we found both lower HC and lower Height Z scores in the ASD group. In sum, the odds of becoming obese due to inadequacy of nutrient intake and gastrointestinal disorders of Egyptian Autistic children is higher than stunting compared to their healthy developing peers.

### Strengths and limitations

This study was carried out at a national scale, directed to a wide geographical area of Egypt, and involved different governorates, presenting a range of eating patterns. Nutritional assessment was done according to RDA, giving extra accuracy of data and results. This study reports a vast study of national interest since it will be helpful for healthcare workers and planners as well.

While the link between factors affecting anthropometry (GIT disturbances as well as nutrition) was established, the link between food selectivity and ASD pathology was not detected by this study.

## Conclusion

Despite the lack of statistical significance for some of the anthropometric indices, the main conclusion was that the BMI-z score for children with ASD was higher and the height-z score was lower than those of their healthy developing peers because children with autism were more likely to evident a sort of protein malnutrition and micronutrient deficiencies compared to their healthy developing peers. Based on our results, although a minority of cases omitted the fruits, vegetables, and protein intake, their intake was infrequent resulting in inadequate intake of vitamins and minerals which may contribute to some autistic behavior. Children with ASD are more selective about foods. Children with ASD have different eating and feeding styles compared to their healthy- developing peers.

### Recommendations

Based on the findings of our study, it is recommended to conduct routine anthropometric measurements for children with ASD at primary health care facilities to evaluate the need for their referral to a specialized dietitian for potentially required intervention on a case-by-case basis. Also focus should be directed towards raising nutritional awareness among caregivers and parents of children with ASD, especially with the success of different community-based interventions for changing population behaviours on their wellbeing for different health aspects in Egypt [75–78].

### Electronic supplementary material

Below is the link to the electronic supplementary material.

Supplementary Material 1

## Data Availability

The datasets used and/or analyzed for the current study are available from the corresponding author upon reasonable request.

## Abbreviations

AOR: Adjusted Odds Ratio
ASD: Autism Spectrum Disorder
BMI: Body Mass Index
CAPMS: Central Agency for Public Mobilization and Statistics
CARS: The Childhood Autism Rating Scale
CI: Confidence Interval
COR: Crude Odds Ratio
DDST: Denver II Developmental Screening Test
DSM-5: Diagnostic and Statistical Manual of Mental Disorders (5th edition)
DRIs: Dietary Reference Intakes
DRI: Daily Recommended intake
DDST: Developmental Screening Test
ERF: Economic Research Forum
FAO: Food and Agriculture Organization
FFQ: Food Frequency Questionnaire
GARS-2: Gilliam Autism Rating Scale
GIT: Gastrointestinal Tract
HC: Head Circumference
MCH: Maternal and Child Health
MUAC: Mid-upper Arm Circumference
M-CHAT-R/F: Modified Checklist for Autism in Toddlers-Revised
MOHP: Ministry of Health and Population
NRC: National Research Centre of Egypt
PHC: Primary Health Care
RDA: Recommended Dietary Allowance
SSB: Sugar-Sweetened Beverages
SPSS: Statistical Package for the Social Sciences
SD: Standard Deviation
UNPF: United Nations Population Fund
UNU: United Nations University
VABSA: Vineland Adaptive Behaviour Scales
WHO: World Health Organization
