# Supplementary material for: The odds of having obesity in Egyptian children with autism spectrum disorders is higher than stunting compared to healthy developing peers: a national survey
Source: BMC Pediatr. 2024 Jul 20;24:465. doi: 10.1186/s12887-024-04934-5 (PMC11264811; doi:10.1186/s12887-024-04934-5)
Supplement: Supplementary file 1 — Supplementary Material 1 [file 12887_2024_4934_MOESM1_ESM.doc]

**S- table 1: List of the randomly targeted MCH and PHC facilities according to the governorates, locality and sociodemographic status for enrollment of Autism children among children aged 3-12 years**

| **serial** | **Gov.** | **classification according to HDI** | **Kesm/Markaz** | **Urban** | **Rural** | |
| --- | --- | --- | --- | --- | --- | --- |
|  |  |  |  | **MCH/PHC Shiakha** | **Local Unit** | **MCH/PHC Village** |
| **1** | **Cairo** | **High** | **AlNozhah** | **Al Hicksit** |  |  |
|  |  | **middle** | **AlSaiedah Zainab** | **Alkabsh** |  |  |
|  |  | **low** | **AlSharabia** | **Al Amiria** |  |  |
| **2** | **Dakhlya** | **High** | **AlSenbelawin** | **Al Sinblaween city** | **Kafr Alruwk** | **Alshalaa** |
|  |  | **middle** | **MietSalsil** | **Mit salsil city** | **Alatihad** | **AlJafara** |
|  |  | **low** | **AlMataria** | **Almataria city** | **Alsafra** | **Al Dahear** |
| **3** | **Gharbia** | **High** | **KafrElZaiat** | **KafrElZaiat** | **Kafour Belshay** | **Qasta** |
|  |  | **middle** | **Samanood** | **Samanood** | **Ziyad’s locality** | **Munshat Nzif** |
|  |  | **Low** | **Markaz of Qutour** | **Qutour** | **Kotour** | **Khabata** |
| **4** | **Fayoum** | **High** | **Markaz of Al Fayoum** | **Alqism rabie** | **Dacia** | **Al Sunbat** |
|  |  | **middle** | **Markaz of**  **Senoures** | **Senoures** | **Terrsa** | **Alzawia El Khadra** |
|  |  | **Low** | **Markaz of Tamiaha** | **Tamiaha** | **Sarsna** | **Kafr Omira** |
| **5** | **Assuit** | **High** | **Hay Shark** | **Alwalidia Alwustania** | **Bani Hussein** | **Musriea** |
|  |  | **middle** | **Al Kousiah** | **Al Kousiah City** | **Mir** | **Bani Hilal** |
|  |  | **Low** | **Al Ghanaiem** | **Al Ghanaiem** | **Alazayiza** | **Al Amri** |
| **6** | **Aswan** | **High** | **Nasr Al Nouba** | **Nasr Al Nouba City** | **Korta** | **Garf Hussein** |
|  |  | **middle** | **Edfo** | **Al-Busaliya Bahri** | **Alramad Albahry** | **Adfu Quabli** |
|  |  | **Low** | **Markaz KoomOmbo** | **KoomOmbo** | **Al Abbasia** | **Sabaa Quabli** |
| **7** | **Damietta** | **High** | **AlRawda** | **AlRawda** | **Hajaja Village** | **Hajaja Village** |
|  |  | **middle** | **Al zarqaa** | **Alsarw** | **Sharmsah** | **Kafr Toqaa** |
|  |  | **Low** | **Kafr-Saad** | **Kafer Albatiykh** | **Kafr Saad Country** | **Nawasiriya village** |
| **8** | **Marsa Matrouh** | **High** | **Marsa Matrouh** | **MarsaMatrouh**  **(Alsanusia & Kilo 4)** | **Alkasr** | **Alkasr** |
|  |  | **middle** | **Al Hamam** | **Al Hamam City** | **Alsalam** | **Alsalam** |
|  |  | **Low** | **AlNajyla** | **AlNajyla** | **Almathany** | **Almathany** |
